# High ^3^He/^4^He ratios in lower East Rift Zone steaming vents precede a new phase of Kilauea 2018 eruption by 8 months

**DOI:** 10.1038/s41598-019-48268-0

**Published:** 2019-08-14

**Authors:** G. M. McMurtry, L. A. Dasilveira, E. L. Horn, J. R. DeLuze, J. E. Blessing

**Affiliations:** 1 School of Ocean and Earth Science and Technology, University of Hawaii, Manoa, Honolulu, HI 96822 USA; 2 School of Ocean and Earth Science and Technology, Hawaii Institute of Geophysics and Planetology, University of Hawaii, Manoa, Honolulu, HI 96822 USA; 3 School of Ocean and Earth Science, University of Southampton, NOCS, Southampton, SO14 3ZH UK; 4 Fusion Energy Solutions of Hawaii, 611 University Ave, Apt. 301, Honolulu, HI 96826 USA; 5 Mass Spectrometry Solutions, MKS Instruments, Inc., 3635 Peterson Way, Santa Clara, CA 95054 USA

**Keywords:** Mass spectrometry, Natural hazards

## Abstract

On May 1, 2018, a magnitude 5.0 earthquake heralded the collapse of the Pu’u O’o Vent on the middle East Rift Zone (ERZ) of Kilauea Volcano, active since 1983. Increased seismicity was recorded on the middle to lower ERZ from April 30 until May 2, 2018. The active lava lakes within both Pu’u O’o Vent and Halema’uma’u Crater began to drain and the summit caldera began to deflate, with the summit collapse ending on August 2, 2018 and lower ERZ eruptive lava activity ending by 4 September 2018. Herein we report on elevated ^3^He/^4^He ratios in steaming vents in the lower ERZ from samples collected in early September 2017. Gas isotopic measurements were made with a new, field-portable He isotope detector capable of sub-daily monitoring of the ^3^He/^4^He ratio. When corrected for air contamination, these values exceed those previously reported for Kilauea by nearly twofold, resembling a purer hotspot plume signature, such as those measured directly over the mantle plume at Loihi Seamount to the SE of Hawaii Island, and in older basalt flows when Kilauea and its sister Hawaiian shield volcanoes were located more directly over the plume. The discovery, which presages the eruption there by more than eight months, suggests that we either sampled a ^3^He/^4^He rich magma already in place in the lower ERZ or a shallow groundwater reservoir in the lower ERZ (Puna district) with anomalously low values of ^4^He relative to their ^3^He/^4^He ratio, similar to previous findings there and suggestive of a previously unknown He isotopic fractionation.

## Introduction

**Figure 1:**
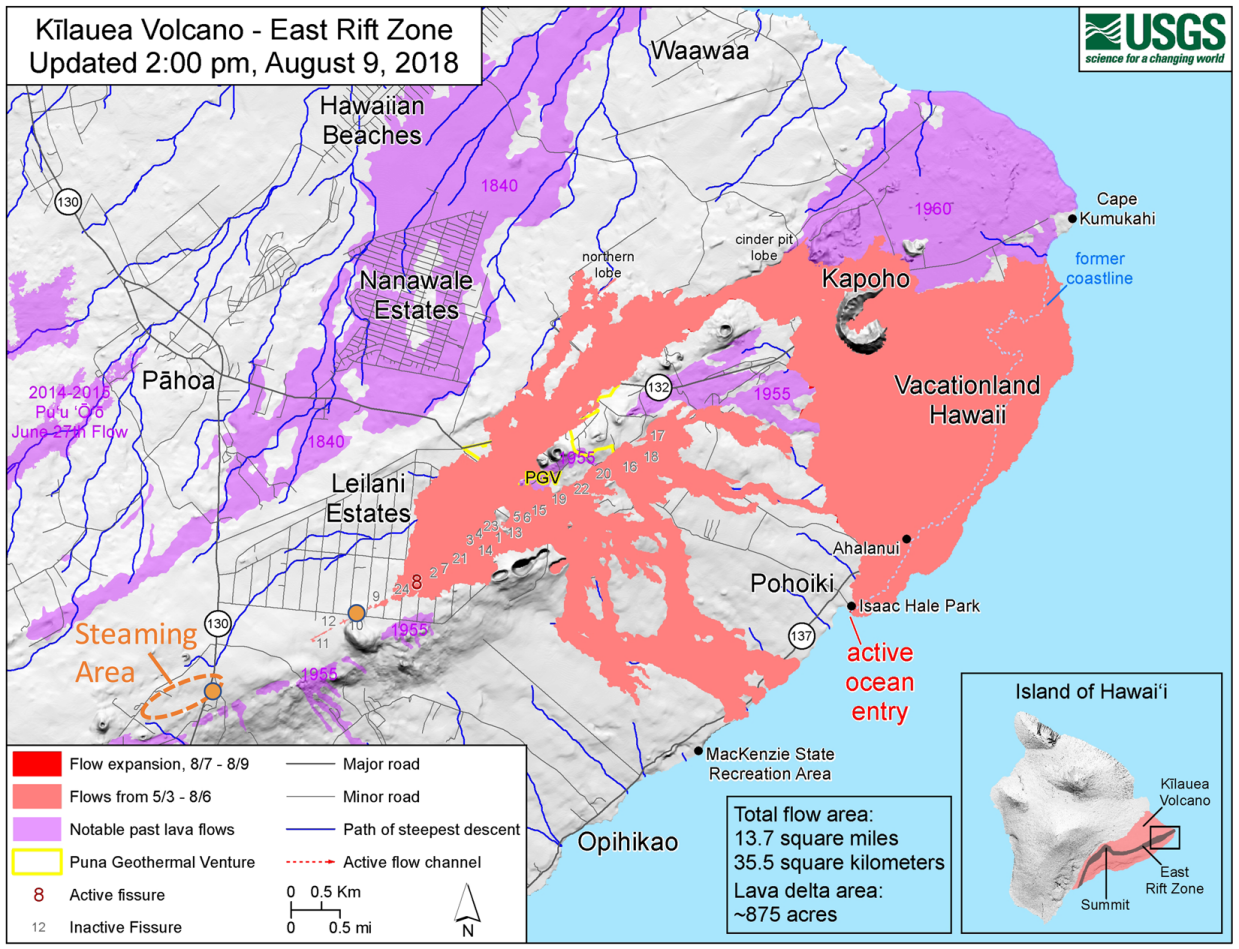
Map of lower East Rift Zone with sample locations in steaming area crossing Highway 130 (orange dot within orange dashed ellipse) and near fissure 10 (orange dot) in the Puna District. Pink and red areas indicate the extent of the 2018 lava flows from 3 May to 9 August, near the end of the extensive lava eruption. Yellow outline shows the boundary of the Puna Geothermal Venture (PGV). The map is one of a series of updates by the U.S. Geological Survey, this version dated from 2:00 PM on August 9, 2018. (Base map and eruption data courtesy of USGS).

Rift-striking cracks began to appear in roads and yards of housing subdivisions within the Puna District on April 30. Initially dismissed as simple dilation along the rift, many of these cracks later evolved from May 3 into the foci of enormous fissure eruptions from 24 new fissures that were mapped by the U.S. Geological Survey. Fissure 8 continued to fire fountain and pour lava into the sea near the former site of Kapoho Bay, now a peninsula. Over 700 houses and nearby agricultural areas were destroyed, including flows covering a portion of the Puna Geothermal Venture (PGV), a 40-MW geothermal power plant near the axis of the ERZ. Observations in early August indicated that this 90-day eruptive phase of Kilauea had abruptly ceased^1^ (Fig. 1).

Helium isotopes are sensitive tracers of volatile provenance and can be used to differentiate between mantle^2^ and crustal derived materials^3^. The ^3^He/^4^He ratio of the upper mantle sampled by mid ocean ridge basalts is ~8 Ra (where Ra = the atmospheric ^3^He/^4^He, 1.4 × 10^−6^), whereas the continental crust has much lower ^3^He/^4^He ratios of ~0.05 Ra due to higher rates of *in situ* radiogenic ^4^He production^4^. Helium isotopes extend as high as 50 Ra in hotspot basalts (gases trapped in glass/vesicles) and in hotspot fumarolic gases and hydrothermal fluids. The higher hotspot values reflect the deep mantle origin of hotspot magma generation^5–8^. As such, geochemists have found it useful to compare and monitor the ^3^He to ^4^He mass abundance ratio in rocks, sediments, free gases and water.

Kilauea’s fluids are characterized by high-^3^He “hotspot” ^3^He/^4^He ratios of between 13.7 and 15.9 Ra^6,9^. These values are lower than those found on Loihi Seamount offshore of Hawaii island, with ratios up to 35 Ra^9–11^, which is consistent with the notion that the Hawaiian plume has undergone extensive degassing prior to incorporation into the source region of Kilauea volcano^6^. Downhole He isotopic analysis of rocks recovered from the Hawaiian Scientific Drilling Project (HSDP) drill hole indicate that in the past, sister Hawaiian shield volcanoes such as Mauna Kea and Mauna Loa possessed high and variable ^3^He/^4^He ratios (from 13 to 25 Ra) when they were closer to the Hawaiian plume, showing more depleted values toward 8 Ra, the upper mantle signature characteristic of Mid-Ocean Ridge Basalt (MORB), as they moved off to the northwest^12^. Indeed, older Kilauea basalts recovered from the summit NSF drill hole show Ra values in olivine phenocrysts of up to 17.1^13^ and those sampled at Pu’u O’o Vent to 16.4 Ra, suggesting that the magma conduit supplying the middle ERZ is separate from that supplying the summit reservoir^14^.

## Methods

In September, 2017, we sampled steaming vents near Highway 130 in the Puna District, in an area to the southwest of Leilani Estates (Fig. 1). Vent gas was collected using a buried inverted funnel and routed through a condensation water trap, using a small thermoelectric refrigerator. Sample gas then entered a custom-built, computer-controlled Vent Gas Purification System (VGPS) consisting of particle filters, high-purity Teflon solenoid valves, a convection pressure sensor, and chemical filter cartridges (Fig. S1, Supplementary Information). The three chemical filter cartridges used contained: (1) indicating calcium sulfate (Drierite™); (2) indicating soda lime; and (3) indicating 4-Å molecular sieve. Sample gas was recirculated through this system to scrub: (1) excess water vapor; (2) carbon dioxide; and (3) small reactive gases such as hydrogen from the vent gas sample. Samples were collected by the VGPS into evacuated He-leak-tight stainless-steel cylinders, either by suction or flow through. Collection involved pumping and recirculation with a compact diaphragm pump specially treated for use with acid gases. Gas recirculation for up to 20 minutes was conducted to better scrub the samples. Two of the three containers used were 1-gallon (3.8 L) capacity stainless steel cylinders with Swagelok™ all-metal bellows valves on each end, called Large Volume Samplers (LVS). One of the samplers, called the “Lincoln Log”, consisted of two joined 6-inch Conflat™ nipples with Swagelok™ all-metal bellows valves on each end and similar 3.8 L capacity.

**Figure 2:**
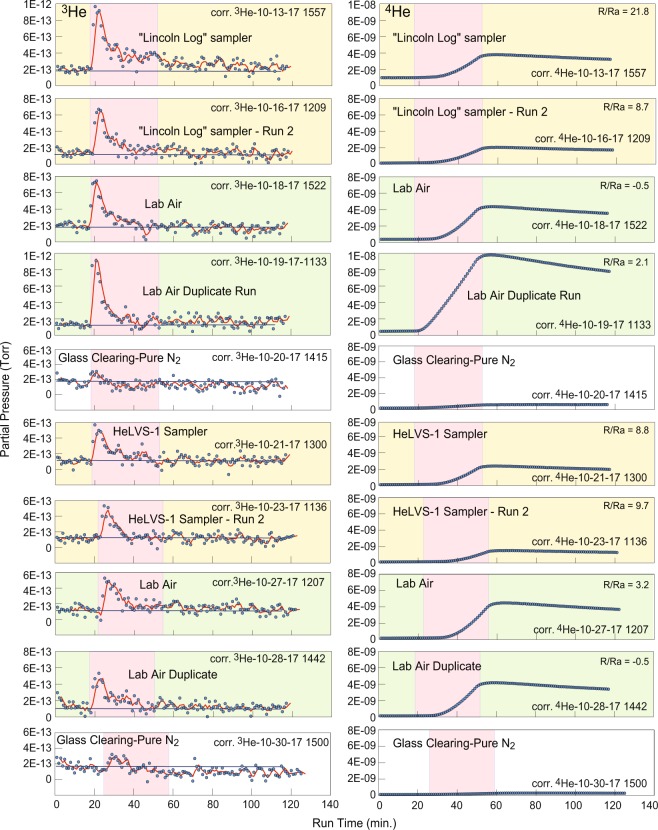
Chronological heat ramp results (top to bottom) showing ^3^He and ^4^He partial pressures (PPs) in Torr versus 57-sec. MS run averages for the lower ERZ sample runs (yellow), along with those for laboratory air (green) and glass clearing blank checks (white), using the “Edward” prototype. Pink zones indicate the 30-minute applied heat ramps (power on to power off) for each sample set. Red curves are 3-point running means through the corrected ^3^He data and heavy black lines indicate mean of the prior ca. 20 background runs before the heat ramp. Sample runs were duplicate analyses with glass clearing under N_2_ between runs. Corrections involve normalization of the calculated PPs for the variable filament emission and electron multiplier gains used in the TIMS method^17^ as described in ref.^16^.

Samples were returned to the lab and run on our prototype portable field helium isotope monitor (Table 1, Fig. 2). Three prototype instruments have been made and utilized to date. “Albert” is a largely manually-operated bench-top unit, with a larger, 2.5-inch diameter cylindrical quartz adapter flange and sample chamber^15,16^. The “Edward” and “Lyle” instruments are identical, relatively compact field-portable versions of the Albert instrument, with smaller 1.5-inch diameter cylindrical quartz adapter flange and sample chamber (Fig. S2, Supplementary Information). Besides the physical difference in high vacuums, the Edward and Lyle units are equipped with DC power supplies instead of a manually-controlled Variac™ autotransformer and use PC-104 based electronic circuit board stacks to control the sampling and data logging via a custom auto-run program written in C++ with Ethernet communications.

**Table 1:** Comparison of measured versus conventional Rc/Ra values, helium isotope monitor.

Sample ID	Prototype Used*	Collection Date	Run Date	R/Ra, inst. corr.**	Measured Rc/Ra***	Conventional Rc/Ra^†^
HESJ	Albert	n/a	8/24/16	21.2	21.2	20.6
	Albert	n/a	9/1/16	20.9	20.9	20.6
	Albert	n/a	10/3/16	23.8	23.8	20.6
	Albert	n/a	10/6/16	21.9	21.9	20.6
	Edward	n/a	9/5/17	20.4	20.4	20.6
	Edward	n/a	9/6/17	24.0 (dup.)	24.0	20.6
	Edward	n/a	9/6/17	21.9 (dup.)	21.9	20.6
Salton Sea	Albert	8/22/14	10/7/16	6.46	6.46	6.4
	Albert	8/22/14	10/8/16	6.35	6.35	6.4
Mammoth HSL	Edward	10/31/17	11/10/17	5.75	5.75	5.1
	Edward	10/31/17	11/17/17	5.91	5.91	5.1
Kilauea Sulfur Bank	Lyle	3/7/18	4/9/18	4.78	11.6^††^	13.5
	Lyle	3/7/18	4/10/18	5.47 (dup.)	13.6^††^	13.5
	Lyle	3/7/18	4/13/18	5.53	13.7^††^	13.5
Kilauea Lower ERZ	Edward	9/9/17	10/16/17	8.7	23.0^†††^	13.7^#^
	Edward	9/9/17	10/21/17	8.8	23.3^†††^	13.7^#^
	Edward	9/9/17	10/23/17	9.7 (dup.)	26.0^†††^	13.7^#^
same site	—	6/9/18	7/13/18	—	—	1.013
1 m away	—	6/9/18	7/13/18	—	—	1.018
Fissure 10 steaming crack	—	6/9/18	7/16/18	—	—	1.034
**Air**						
Lab Air Average	Edward, n = 8	10-11/17	10-11/17	0.36 (3.1)^##^	0.36	1.0
Lab Air Average	Lyle, n = 6	3-4/18	3-4/18	−0.35 (1.5)^##^	−0.35	1.0
Lab Air Average	Albert, n = 5	8-9/16	8-9/16	2.24 (2.5)^##^	2.24	1.0
Air Ave., Mammoth HSL	Lyle, n = 52	6-7/18	6-7/18	0.40 (0.69)^##^	0.40	1.0

*Three prototype instruments have been made and utilized to date. Albert is a bench-top unit, with a larger, 2.5-inch diameter cylindrical quartz adapter flange and sample chamber. Edward and Lyle are intended as identical, relatively compact field-portable units with smaller 1.5-inch diameter cylindrical quartz adapter flange and sample chamber^15,16^.

**We use a linear correction of y’ = (y/1.83) − 2.45 to correct the net integral results of the heat ramps, set to 100 sample scan averages past the beginning of the ramp. This linear correction was produced by an earlier regression analysis using the HESJ, Salton Sea, and Lab Air as standards run on Albert.

***Air contamination corrections were made based upon assumed dry air values for N_2_, O_2_ and Ar of 78.09, 20.95, and 0.93% (source: ref.^27^), and using relative Ar concentrations.

^†^Mean value for HESJ^28^. Salton Sea mud pots from D. Hilton lab, SIO. Mammoth HSL, Kilauea Sulfur Bank and Lower ERZ values from USGS Noble Gas Lab, Denver Federal Center, A. Hunt, analyst.

^††^Air corrected values using measured N_2_, O_2_ and Ar values of 59.3, 12.3 and 0.6% for sample SB-18, Friedman well, Sulfur Bank, collected on 7 March 2018, D. Bergfeld, USGS, analyst. Maximum vent temperature varied from 73° to 105 °C during collection.

^†††^Air corrected values using minimum N_2_, O_2_ and Ar values of 59.3, 12.3 and 0.6% as for sample SB-18. Maximum vent temperature ranged from 72° to 74.5 °C during collection, 9 September 2017.

^#^Value measured from nearly pure vent gas collected at Jagger well, Sulfur Bank on 7 March 2018, A. Hunt, USGS, analyst.

^##^Mean and one-sigma standard deviation (in parentheses).

Summarized, the operations include volume expansion of collected sample gas into a cylindrical sample chamber evacuated to a pressure of about 2 Torr. Nitrogen pressurization was used to raise the sample pressure back to 1-atm total pressure before the heat ramp, which was done to keep all sample runs at constant pressure. Inside the chamber, a cylindrical high-purity quartz glass adapter flange is heated to over 400 °C, allowing helium, plus some hydrogen, neon, and argon to pass into a high vacuum chamber (HVC). An MKS Instruments Microvision 2 high-resolution quadrupole mass spectrometer analyzes the HVC contents by running a preprogramed sequence of varying the ionization potential and electron multiplier gain while rapidly scanning through the 1 to 6 amu (Dalton) range. Varying the electron multiplier detector gain provides sufficient instrument dynamic range to sequentially detect both the natural ^3^He and ^4^He abundance over their large dynamic concentration range (Fig. 2). These processes allow utilization of the threshold ionization MS (TIMS) method of Davies *et al*.^17^ so that isobaric interference by HD and any similar isobars (H_3_, ^3^H) can be corrected from the mass-3 peak to reveal the ^3^He isobar. A discussion of the reliability of this isobaric separation can be found in the Supplementary Information of ref.^16^. We routinely collect over 100 approximately 1-minute scan averages during a sample heat ramp. Post-processed data are converted from binary to text files and analyzed in Microsoft Excel. Collected gases are purged from the high vacuum by exposure to noble ion and non-evaporable getter pumps (c.f., McMurtry *et al*.^15,16^). Operation of this monitor is more fully described in McMurtry *et al*.^16^.

The results of the Lower ERZ sample heat ramps conducted in October 2017 are presented in Fig. 2. The ^3^He and ^4^He partial pressures (PP, in Torr) of gas samples (yellow highlight) are stacked chronologically along with lab air runs (green highlight) and glass clearing runs (no highlight color). The 2-hour sample heat ramps consist of a predetermined cycle from ambient lab temperature (ca. 22 °C) to ca. 400 °C that was held for 30 minutes before removing power and cooling back to ambient room temperature, with an initial 20 minutes of background acquisition. After all sample runs, the glass is cleared of the previous sample by evacuation of the sample chamber to under 2 Torr pressure and running the same temperature ramp under 1-atm of pure tank nitrogen for 90 minutes at the maximum power setting, with simultaneous or subsequent exposure to a noble diode ion pump. The glass clearing runs are made after purging the sample chamber under nitrogen at 1-atm pressure and running under the same conditions as the gas samples and lab air. These serve as a blank check for the sample runs.

The lab air R/Ra shown in Fig. 2 varies from −0.5 to 3.2 with a mean of 1.1 ± 1.9 (n = 4). This degree of variation reflects, partially, the limit of sensitivity of the current instrument as it approaches the 1.0 R/Ra level of ambient air. (Negative R/Ra values can result when the background is higher than the ramp signal due to suppression of the ^3^He PP response by that from the more abundant ^4^He PP^16^). However, the R/Ra of the lower ERZ samples are significantly higher. The heavy black lines of the ^3^He PPs in Fig. 2 indicate the mean of the prior ca. 20 background runs before the heat ramp, which is 2.0E-13 Torr or somewhat lower for ^3^He. The early heat ramp peaks of the vent gas samples and air, caused by a differential diffusion effect for helium isotopes and described more fully elsewhere^16^ are all in excess of this background. Variations in the R/Ra between the lab air and vent gas samples are primarily a result of variable ^4^He PPs relative to those of ^3^He, with the air runs showing greater ^4^He PPs (Fig. 2).

Collected gas pressures were 760, 633, and 327 Torr for the Lincoln Log, LVS-1 and LVS-2 samplers. The total collected pressure of 327 Torr in the LVS-2 sampler was considered too low to reliably measure isotopic ratios by vacuum expansion. The low pressure was a result of a too short filling time for an evacuated sample chamber with very low conductance valves. Of the lower ERZ samples, the first run of the Lincoln Log sampler displays a very large R/Ra value of 21.8 (Fig. 2). We rejected this higher result because for the first run the instrument had sat for a prolonged period with its sample chamber under nitrogen atmosphere where lab air may have entered and enriched the glass in ^3^He at low temperature, which is more fully described in McMurtry *et al*.^16^.

## Results and Discussion

**Figure 3:**
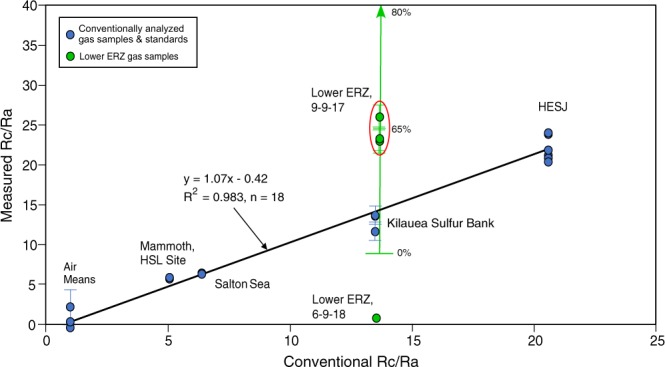
Plot of known (air) or conventionally-determined value versus measured Rc/Ra from this study, using the field portable helium isotope monitor instrument^15,16^. Bold line denotes linear regression fit to all data except those samples from the lower ERZ. Green data points are analyses of lower ERZ samples collected in September 2017 (n = 3) and the mean of 5 conventional analyses (Rc/Ra = 1.06 ± 0.09) from samples collected at the same sampling site in June 2018 (two duplicates), plus one from the nearby fissure 10 in Leilani Estates. For comparison, these data are plotted against the expected Rc/Ra from Sulfur Bank, on the Kilauea summit. Green line with arrow indicates the range of possible air contamination, as percentages, for the lower ERZ samples collected in September 2017. Red oval denotes the likely minimum air correction for the lower ERZ steaming vent samples.

The results of our September 2017 sampling on the lower ERZ of Kilauea are presented in Table 1 and Fig. 3, along with sampling at Sulfur Bank on the Kilauea summit, together with those of standards and measured natural gases, plus laboratory air used in the calibration of our monitoring instruments. Samples of vent gas taken to correct for air contamination were not collected in the lower ERZ at that time, but it is reasonable to assume that these lower temperature vents were at least as contaminated as those collected by us at the Sulfur Bank “Friedman” or “New Well” site on the Kilauea summit in March 2018 (Table 1; ref.^18^). Here we compare the similar vent temperatures and open, physical characteristics of these steaming vents, where subterranean air contamination is often present. Lesser air contamination is possible, but unlikely. More air contamination would only increase the lower ERZ R/Ra values, as shown in Fig. 3.

Of the conventionally analyzed samples used in the regression analysis (Fig. 3), the HESJ and Salton Sea samples were from an artificial gas mixture and a natural vent gas that were purified in the lab, respectively, and therefore needed no correction for air contamination. The Mammoth HSL site samples were run as consecutive splits of an 85% carbon dioxide soil gas collected in the two LVS samplers but not purified in the VGPS beforehand. The Kilauea Sulfur Bank samples were run through the VGPS on site and collected in the LVS and Lincoln Log samplers. These samples were corrected for air contamination using argon concentrations measured from sample splits. The conventionally analyzed splits of these samples (e.g., those run on high-resolution sector noble gas MS after sample purification steps) were corrected for air contamination using their Ne/He data. Although these prototype instruments are equipped with a second MS that is capable of measuring ^20^Ne and ^40^Ar PP, which are also known to diffuse through the glass, this MS was not recording properly when the samples were measured on the Edward prototype. Comparison with a nearly pure sample of vent gas collected at Sulfur Bank, from the Jagger Well site nearby to the Friedman Well (Table 1), suggests that these air corrections were accurate to within 2%.

For these air corrections, we assumed a two-component mixing of pure fumarole gas and ambient air. Using argon as a conservative air signature gas, we calculate an algebraic equation, using a value of 0.0024% on a dry basis for Ar from a 308 °C fumarole sample collected at the base of the Halema’uma’u Crater (Sample KV97-3) in May 1997^19^. For dry air, we used a value of 0.93% (Table 1). The mixing equation for the Kilauea Sulfur Bank results is as follows:

$$\begin{array}{rcl}({\rm{x}})\,{\rm{Ar}}\,({\rm{pure}}\,{\rm{fumarole}})+(1-{\rm{x}}){\rm{Ar}}({\rm{air}}) & = & {\rm{Ar}}\,({\rm{measured}});0.0024{\rm{x}}+(1-{\rm{x}})(0.93)\\  & = & 0.60;\,{\rm{x}}=0.356\end{array}$$

where x = the fumarole gas fraction.

To calculate the Rc/Ra values from the instrument corrected R/Ra values in Table 1 (R/Ra**), we assume the values for air Ra = 1.0, yielding:

$$\begin{array}{c}{\rm{R}}/{\rm{Ra}}({\rm{x}})+{\rm{Ra}}(1-{\rm{x}})={\rm{Rc}}/{{\rm{Ra}}}^{\ast \ast }\\ {\rm{Rc}}/{\rm{Ra}}(0.356)+(1.0)(0.644)=(1.0)4.78\\ {\rm{Rc}}/{\rm{Ra}}={\bf{11}}.{\bf{6}}\,{\rm{for}}\,{\rm{sample}}\,{\rm{HeLVS}}-2,\,{\rm{run}}\,1\end{array}$$

$$\begin{array}{c}{\rm{Rc}}/{\rm{Ra}}(0.356)+(1.0)(0.644)=(1.0)5.47\\ {\rm{Rc}}/{\rm{Ra}}={\bf{13}}.{\bf{6}}\,{\rm{for}}\,{\rm{sample}}\,{\rm{HeLVS}}-2,\,{\rm{run}}\,2\end{array}$$

$$\begin{array}{c}{\rm{Rc}}/{\rm{Ra}}(0.356)+(1.0)(0.644)=(1.0)5.52\\ {\rm{Rc}}/{\rm{Ra}}={\bf{13}}.{\bf{7}}\,{\rm{for}}\,{\rm{sample}}\, \mbox{``} {\rm{Lincoln}}\,\mathrm{Log}\mbox{''},{\rm{run}}\,1\end{array}$$

The estimated error based upon duplicate runs = 14.6%, which are reflected in the error bars in Fig. 3. Also, a much higher or lower calculated Rc/Ra for Kilauea Sulfur Bank would detract from the goodness of fit of the linear regression analysis in Fig. 3, suggesting that the value measured from the Jagger Well site was accurate and representative at that time.

We revisited the lower ERZ sampling site on 9 June 2018 and found the vent temperature had dropped from a maximum of 74.5 °C to a maximum of only 63 °C. Two duplicate sets of samples taken in copper tubes for helium isotopes at this site plus one from a higher temperature steaming crack in nearby Leilani Estates (max. temperature of 126 °C) yielded a mean Rc/Ra value of 1.06 ± 0.09 (n = 5, A. Hunt, USGS, analyst). Although new, copiously steaming cracks had crossed Highway 130 about 100 m to the northeast, the original sampling site had cooled significantly and showed little indication of a volcanic gas component at that time. The lower ERZ is not generally devoid of hotspot Rc/Ra values, however, as deep geothermal wells drilled there tap high-temperature fluids with R/Ra values of up to 12.1 in steam collected at the top of the well^20^. Later, Rc/Ra values of 14.3 to 15.1 were measured in the deep PGV production wells (n = 5), with Rc/Ra values of 5.3 to 10.8 (n = 3) reported in the shallow groundwater there [21; see Fig. S3, Supplementary Information].

Using the likely minimum air correction yields Rc/Ra values of 23 to 26, which are reasonable for the Hawaiian hotspot plume, but quite unexpected for Kilauea, unless the new eruption beginning on 3 May tapped a relatively un-degassed portion of the plume. If so, then we inadvertently discovered a ^3^He/^4^He rich magma already in place in the lower ERZ at least eight months prior to the eruption there. The lower vent temperatures and atmospheric Rc/Ra values measured in the lower ERZ in June 2018 further suggest that the September 2017 magmatic gas signal was short-lived but could be preserved in the earlier lavas erupted there. Kurz *et al*.^12^ found ^3^He/^4^He spikes in the mostly submarine lavas of Mauna Kea had a modeled duration of 15 ± 9 Ka, but some of the twelve excursions measured represented single intrusion events and lava flows. They argued that these spikes represent undegassed mantle plume material and are found in relatively small, shallow magma chambers such as a young Mauna Kea and present-day Kilauea.

A competing but equally intriguing hypothesis is we sampled the same or similar shallow groundwater reservoir in Puna as that sampled by Fercho *et al*.^21^. Those waters have anomalously low values of ^4^He relative to their ^3^He/^4^He ratio, a qualitatively similar characteristic of the samples we analyzed in the lower ERZ steaming vents (Fig. 2). The samples from Fercho *et al*. do not follow an expected dilution curve between the high-temperature reservoir and air-saturated water endmembers, as opposed to other rift zone waters analyzed by them from Maui and Hawaii islands [21; see Fig. S3, Supplementary Information]. These data suggest there is a previously overlooked ^3^He/^4^He fractionation effect of helium dissolved in waters that probably underwent boiling and phase separation before dilution with meteoric groundwater. If so, this effect would have broad implications for any previous work that assumed that there was no or little fractionation of these isotopes in such conditions.

Adding credence to our analytical results on the lower ERZ samples, the same He isotope monitor prototype run under the same lab conditions was able to determine the Rc/Ra value at Horseshoe Lake (HSL) site on Mammoth Mountain, CA to within 16% too high of the conventional determination (n = 2, Table 1). The companion field prototype was later able to determine the Rc/Ra value at the Kilauea Sulfur Bank field to within 1.5% of the conventional determination on two runs, and had a worst-case match of 13% too low (Table 1).

## Conclusions

Our results promote the notion that continuous monitoring of ^3^He/^4^He in vent gases can be an effective alert for future eruptions at Kilauea and elsewhere, especially where previous conventional sampling revealed a positive correlation between elevated ^3^He/^4^He and volcanic unrest^22–25^. In September 2017 when the gas samples were collected, the instrumentation was new and operating in a sample return mode to our laboratory, with similar VGPS sampling in the field continuing through the Kilauea Sulfur Bank operations in March 2018. It is apparent that the analytical method is presently less precise as the R/Ra values approach those of air. It does respond well and linearly above that current threshold, however, and we are actively pursuing ways to improve the sensitivity so that the monitor can also be applied in continental crust environments, where many major faults as well as active volcanoes are found.

These results for the lower ERZ have further, significant implications for the mode of He and ^3^He/^4^He enrichments in vent gases at Kilauea and elsewhere. If the favored heterogeneous magma hypothesis is correct, magma bodies or deep fluids influenced by them [e.g., ref.^26^ can extend down the length of the ERZ with Loihi-type ^3^He/^4^He enrichments, heralding a new eruptive phase of the volcano, which has tapped the deep, relatively undegassed plume. If the ^3^He/^4^He fractionation hypothesis is correct, then groundwater in the Puna area of the lower ERZ contains evidence for a previously unknown He isotopic fractionation associated with boiling and phase separation prior to dilution with meteoric waters. The signal is apparently transient and not reflected in subsequent degassing, and may or may not be recorded in the later erupted lava flows there. We caught this signal by serendipitous sampling, while testing a new field instrument. However, the nature of the enrichment displayed demonstrates the need for deployment of similar field instruments that can catch such transitory signals and allow collection of potential precursory data, which can be acted upon in the future.

## Supplementary information

Supplementary Information

## Data Availability

The datasets generated during and/or analyzed during the current study are available from the corresponding author on reasonable request.
