# Supplementary material for: High 3He/4He ratios in lower East Rift Zone steaming vents precede a new phase of Kilauea 2018 eruption by 8 months
Source: Sci Rep. 2019 Aug 14;9:11860. doi: 10.1038/s41598-019-48268-0 (PMC6694148; doi:10.1038/s41598-019-48268-0)
Supplement: Supplementary file 1 — Supplementary Information [file 41598_2019_48268_MOESM1_ESM.pdf]

Supplementary Information

High $^3He/^4He$ ratios in lower East Rift Zone steaming vents precede a new phase of Kilauea 2018 eruption by 8 months

G. M. McMurtry[1,*], L. A. Dasilveira[2], E. L. Horn[3], J. R. DeLuze[4], and J. E. Blessing[5]

1. School of Ocean and Earth Science and Technology, University of Hawaii, Manoa Honolulu, HI 96822 USA mcmurtry@hawaii.edu

2. School of Ocean and Earth Science and Technology, Hawaii Institute of Geophysics and Planetology, University of Hawaii, Manoa Honolulu, HI 96822 USA luis@hawaii.edu

3. School of Ocean and Earth Science, University of Southampton, NOCS, Southampton, SO14 3ZH UK e.l.horn@soton.ac.uk

4. Fusion Energy Solutions of Hawaii, 611 University Ave, Apt. 301, Honolulu, HI 96826 USA jrdeluze@icloud.com

5. Mass Spectrometry Solutions, MKS Instruments, Inc., 3635 Peterson Way, Santa Clara, CA 95054 USA James_Blessing@mksinst.com

* Correspondence to: Gary McMurtry (mcmurtry@hawaii.edu)

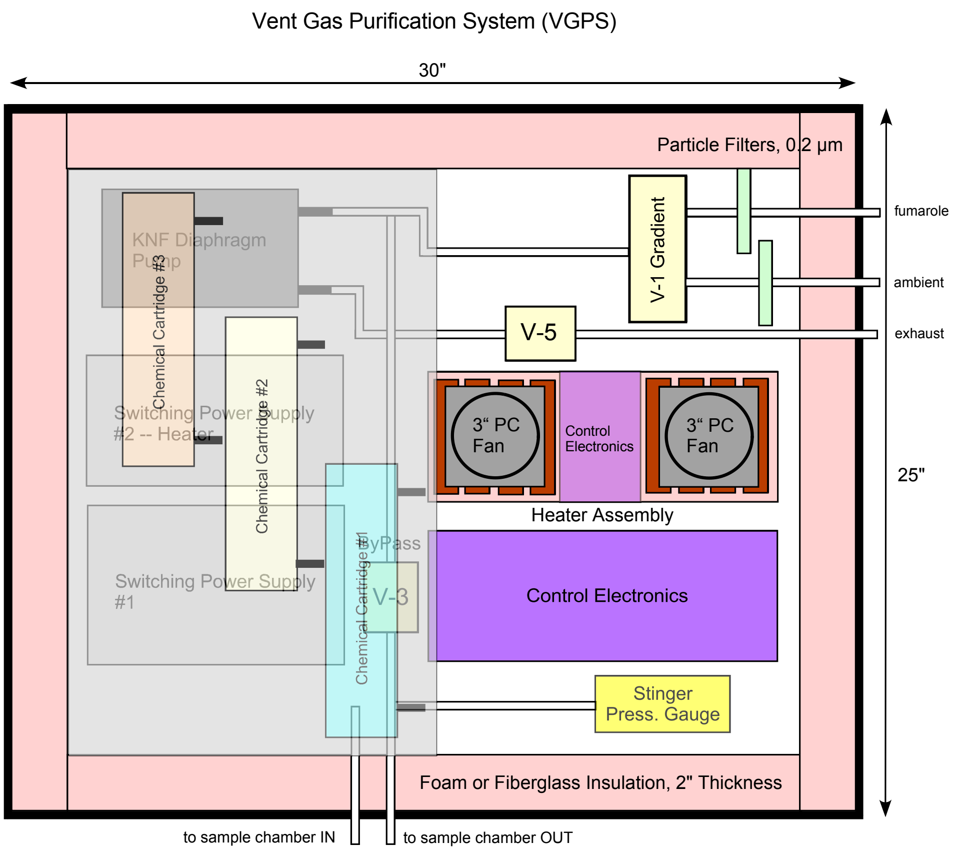

Fig. S1. Schematic diagram of the Vent Gas Purification System (VGPS). All components fit within a portable Pelican™ case. Three removable chemical cartridges clamp onto the top layer, with two DC power supplies and a diaphragm roughing pump located below. Internal Teflon™ solenoid valves direct gas flow. Pressure is measured with an InstruTec™ Stinger convection gauge. A custom command electronics board relays valve openings, temperatures, pressures and roughing pump on/off via a Rabbit™ microprocessor. A small heater assembly is used for extremely cold environments.

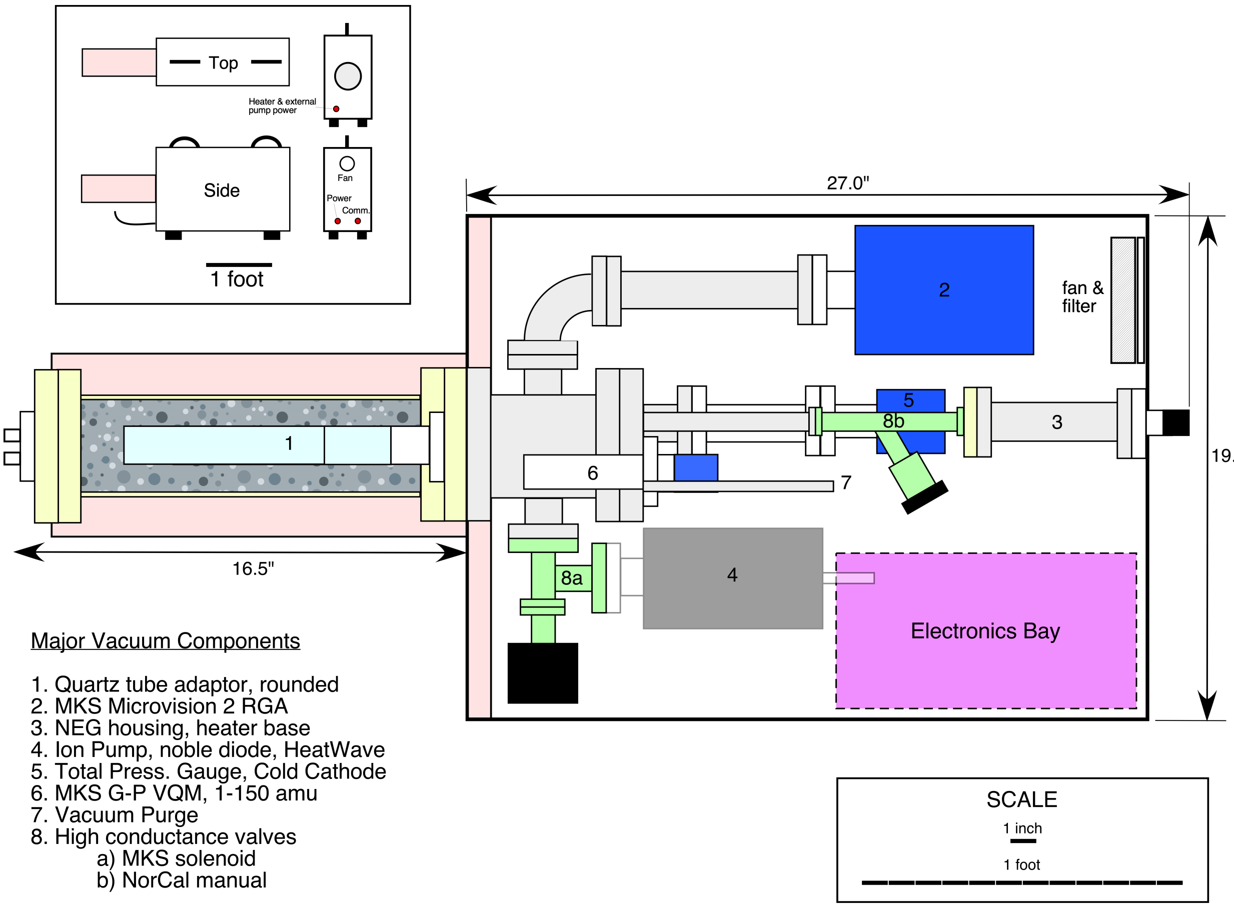

Fig. S2. Schematic diagram of portable field helium isotope detector, showing major vacuum components. A detailed description of the working procedure for this instrument can be found in McMurtry et al. [16].

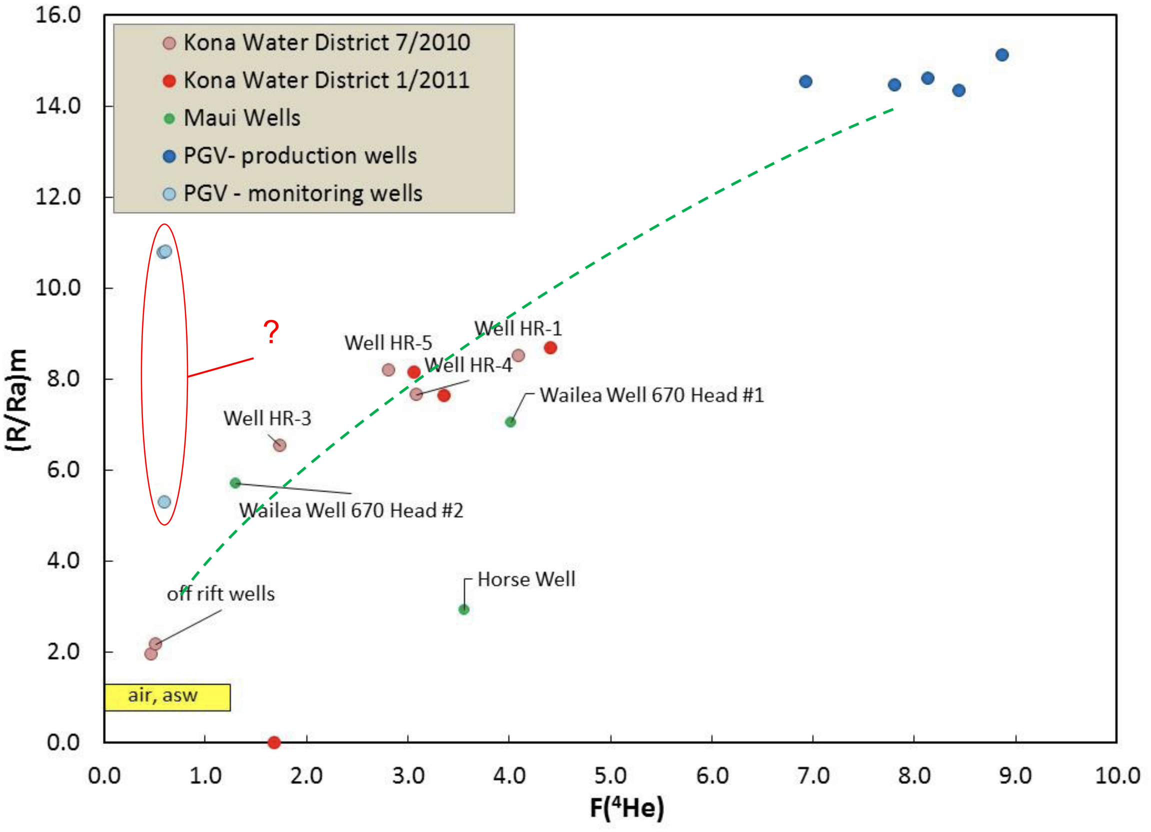

Fig. S3. Measured air-corrected helium isotopic compositions [(R/Ra)c] in waters as a function of the ^{4}He enrichment factor [F(^{4}He)], where F[^{4}He] = (^{4}He/^{36}Ar)mantle/(^{4}He/^{36}Ar)air. Data trend for Hualalai and Wailea samples is consistent with mixing of magmatic helium enriched components with air-saturated water (dashed green line). Anomalous groundwater samples (n=3) from PGV monitoring wells are circled in red. Figure modified from Fercho et al. [21].
